# Biological responses to biomaterials: a review

**DOI:** 10.1590/1414-431X2025e14599

**Published:** 2025-05-09

**Authors:** Y.V.R. Gomes, A.A. Tavares, R.C. Barbosa, A.F. Tomaz, W.J.B. Sousa, L.C.C. Oliveira, S.M.L. Silva, M.V.L. Fook

**Affiliations:** 1 Programa de Pós-Graduação em Ciência e Engenharia de Materiais, Departamento de Engenharia de Materiais, Universidade Federal de Campina Grande, Campina Grande, PB, Brasil; 2 Departamento de Fisioterapia, Universidade Estadual da Paraíba, Campina Grande, PB, Brasil

**Keywords:** Biocompatibility, Regeneration, Immune response, Inflammation

## Abstract

Biomaterials stimulate diverse biological responses, including inflammation, wound healing, foreign body reactions, and fibrous encapsulation, all critical for evaluating biocompatibility and effectiveness. These responses are influenced by the material's physicochemical and biological properties, such as composition, texture, and surface characteristics. Adverse reactions, such as severe inflammation or fibrous encapsulation, can hinder tissue integration, jeopardizing patient health and increasing healthcare costs. This review aimed to summarize the current scientific evidence on biological responses to biomaterials. A systematic search was conducted through multiple databases (VHL, PubMed, SCOPUS, EMBASE, and Web of Science) including *in vitro* and *in vivo* studies that compared biomaterial interactions with the natural immune response (innate and adaptive). From the 791 articles identified, 25 met strict inclusion criteria. These studies revealed variations in immune responses and material surface characteristics, highlighting advancements made to enhance tissue integration. Bioactive materials demonstrated greater potential for tissue regeneration, while inert materials triggered moderate inflammatory reactions. This variability emphasizes the need for a personalized biomaterial selection, considering both short-term biocompatibility and long-term tissue functionality. This review underscores the importance of comprehensive evaluation to optimize biomaterial performance in clinical applications.

## Introduction

Biomaterials encompass a diverse range of materials employed in various biomedical applications, such as medical implants, diagnostic devices, drug delivery systems, and tissue engineering (1). Specifically engineered to interact with biological systems, these materials provide essential structural support, promote tissue regeneration, and modify biological responses to achieve targeted therapeutic outcomes (2). Despite their therapeutic potential, the introduction of biomaterials into the human body elicits a series of complex and interdependent biological responses that significantly influence their biocompatibility and overall clinical performance (3).

Upon implantation, biomaterials initiate a cascade of biological events that commence with interactions between the biomaterial and adjacent cells and tissues. The inflammatory response is a primary reaction of the immune system to foreign bodies, varying in intensity and duration based on factors such as the biomaterial's chemical composition, surface topography, mechanical properties, and the individual characteristics of each patient (4,5). Specifically, the surface properties of biomaterials can trigger inflammation through the activation of factor XII, leading to the fibrinolytic cascade and activation of the complement system, both of which play crucial roles in mediating the inflammatory response. Although there are strategies to design biomaterials with optimized physical and chemical properties to modulate this response, a truly “inert” biomaterial remains elusive (6).

The biological response to the implantation of a biomaterial is a complex process that can result in both rejection and implant success. Initially, an acute inflammatory response occurs, followed by the formation of granulation tissue and, eventually, tissue remodeling. Implant rejection can be triggered by an exacerbated immune response, fibrosis formation, or infection. On the other hand, implant success is associated with adequate integration of the biomaterial with the host tissue, minimization of the chronic inflammatory response, and promotion of tissue regeneration (7). Advances in the types of biomaterials, such as biodegradable polymers, bioactive ceramics, and composites, have shown promising results in preclinical and clinical studies, standing out for their biocompatibility and ability to support tissue regeneration. These advances not only expand therapeutic options, but also offer new perspectives for the development of more effective and durable implants (8,9).

The introduction of implants into the body triggers a series of intricate reactions within the immune system. The activation of immune cells such as macrophages and lymphocytes occurs in response to the presence of foreign bodies. These cells are recruited to the implant site where they engage in phagocytosis to eliminate foreign materials (10,11). This immune response involves the release of inflammatory cytokines, including interleukins and tumor necrosis factors, which can initiate a localized inflammatory reaction. Under certain circumstances, this response may become dysregulated, potentially leading to allergic or autoimmune reactions against the host's own tissues (12).

This review aims to provide a comprehensive overview of biological responses to biomaterials, highlighting the critical importance of understanding these interactions for the development of safer and more effective materials. The insights gained from this analysis are vital not only for the advancement of regenerative medicine and tissue replacement therapies, but also for future research in various healthcare applications such as breast implants, dental implants, orthopedics, wound dressings, controlled drug delivery systems, and others. By elucidating the intricate interactions between biomaterials and biological systems, this study underscores the importance of biological responses in the design and evaluation of biomedical products and encourages continued research in the field of human health.

## Methodology

### Protocol and registration

The study was performed based on the 2020 PRISMA (Preferred Reporting Items for Systematic Reviews and Meta-Analyses) statement (13). The review protocol was officially registered with the Open Science Framework (OSF) under the identifier (osf.io/em4xt).

### Eligibility criteria

#### Inclusion criteria

This systematic review employed the PICO framework, comprising Population, Intervention, Comparators, and Outcome (14), to establish the criteria for study inclusion. Eligible studies were those that examined the biological response (outcome) to biomaterials (intervention) in relation to the natural immune response (both innate and adaptive) to foreign bodies (comparator). The review considered both *in vitro* and *in vivo* models, encompassing preclinical and clinical research (population).

#### Exclusion criteria

Studies that did not involve biomaterials as part of the intervention and did not address immune responses (innate or adaptive) as an outcome were excluded from the review. Additionally, review articles, book chapters, theses, letters, personal opinions, conference abstracts, and patents were not considered.

### Search sources and strategy

Distinct search strategies were used for each database consulted during the literature search, which included the Virtual Health Library (VHL), PubMed, SCOPUS, EMBASE, and Web of Science (see Supplementary Table S1). Search terms were selected based on Medical Subject Headings (MeSH), Health Sciences Descriptors (DeCS), and Elsevier's Thesaurus for Biological Sciences (Emtree) for each element of PICO. The main terms (“Prostheses and Implants”, “Regeneration”, “Immunological Factors”, and “Biocompatible Materials”) and their synonyms were combined using the Boolean operators OR and AND. This research was conducted in 2024, without imposing any restrictions on language or publication date. Duplicate references were eliminated using reference management software (EndNote X9, USA).

### Study selection

The article selection process consisted of two distinct phases to ensure each study's alignment with the established inclusion and exclusion criteria. Initially, two authors (A.A.T. and R.C.B.) reviewed the titles and abstracts of all identified studies using Rayyan (https://www.rayyan.ai), an online tool that streamlines the screening process for systematic reviews. In the subsequent phase, these authors conducted a comprehensive full-text evaluation of the studies shortlisted from the first phase, excluding those that did not meet the inclusion criteria (see Supplementary Table S2). Any disagreements between the authors were resolved through discussion with a third author. Following the completion of the selection process, relevant data from the studies, including author, year, country, study model, type of biomaterial, and immunological response, were collected and organized in a tabular format.

## Results and Discussion

### Study selection

**Figure 1:**
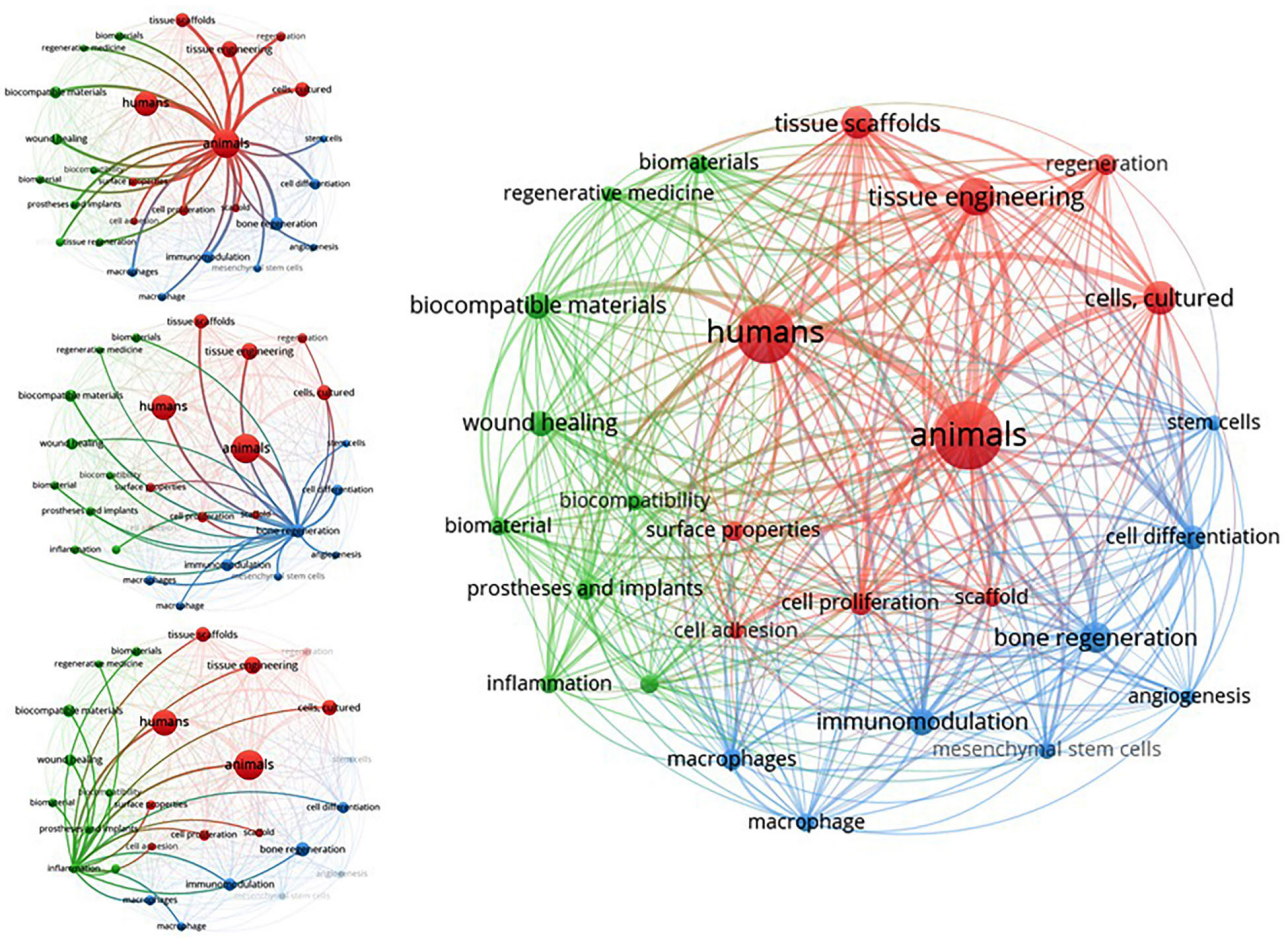
Network visualization showing the co-occurrence and connectivity of terms identified in the “Titles, Keywords, and Abstracts” of publications retrieved from the databases. The analysis was performed using VOSviewer 1.6.20 software (2023) with a “full counting” configuration and a minimum of 22 occurrences. The colors represent groups of correlated terms identified through cluster analysis: Group 1 (Red) with 10 items, Group 2 (Blue) with 8 items, and Group 3 (Green) with 9 items. Sources: VHL, PubMed, SCOPUS, EMBASE, and Web of Science (2024).

The literature search was performed on August 29, 2024, which yielded a total of 791 publications. The analysis of recurring terms in the dataset revealed that the academic community primarily focuses on topics such as animals, humans, biocompatible materials, tissue engineering, biocompatibility, immunomodulation, wound healing, inflammation, cell differentiation, prosthetics, and implants. Figure 1 summarizes these findings by presenting the bibliometric network of terms appearing at least 22 times in the titles, keywords, and abstracts of the documents. The visualization, configured with “co-occurrence”, “full counting”, and “keywords”, indicates the most frequent terms by larger spheres.

**Figure 2:**
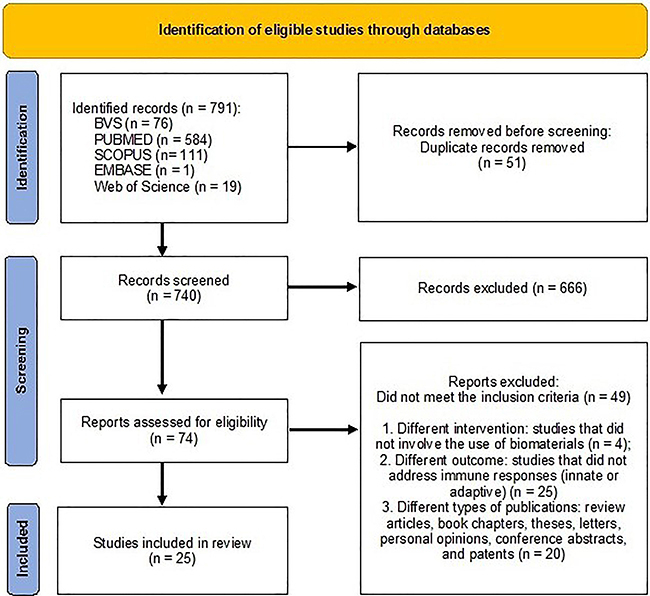
Flow diagram of the literature search and selection criteria based on PRISMA guidelines (15). The diagram illustrates the systematic process of study selection. A total of 791 records were retrieved from five databases, and 51 duplicates were removed, leaving 740 records for screening. Of these, 666 were excluded for irrelevance, and 74 full-text articles were assessed for eligibility. Among them, 49 studies were excluded due to inappropriate interventions (n=4), irrelevant outcomes (n=25), or unsuitable publication types (n=20). Ultimately, 25 studies met the inclusion criteria and were included in the review.

The study selection process resulted in 25 articles being included for analysis in this review, following a thorough screening and application of predefined criteria. Figure 2 outlines this process (15), starting with the retrieval of 791 studies from various databases: 76 from VHL, 584 from PubMed, 1 from Embase, 111 from Scopus, and 19 from Web of Science. After removing duplicates, 740 studies were assessed, leading to the exclusion of 666 based on titles and abstracts. A full-text review of the remaining 74 articles led to the exclusion of 49 studies.

### Characteristics of included studies (n=25)

The studies included in this review specifically evaluated the biological response to various biomaterials. A summary of the main characteristics of these studies is presented in Supplementary Table S3 (16-40). These investigations focused on host response to different biomaterials implanted in animal models, with an emphasis on immunological interactions and healing processes. The materials tested encompassed acellular dermal matrices, polymers, ceramics, composites derived from porcine, human, and primate sources, as well as graphene and collagen-chitosan scaffolds. Key research areas included inflammation, angiogenesis, cell repopulation, macrophage activation, and phenotypic polarization (M1/M2), in addition to the effects of decellularization and the chemical composition of the biomaterials. The findings revealed significant variations in the inflammatory response and integration of the biomaterials, underscoring the critical role of structural properties and processing methods in determining the clinical efficacy and immunological acceptance of these implants.

Most of the included studies used *in vivo* models, predominantly in rodents and primates, while some combined *in vivo* and *in vitro* investigations to evaluate different aspects of the interactions between biomaterials and biological tissues. These reported varied immunological responses, often associated with the presence of inflammatory cells and the release of cytokines, the magnitude of which depended on the type of biomaterial used. For example, the studies by Sandor et al. (16) and Xu et al. (17) highlighted intense cellular infiltration and significant antibody production when using biological meshes derived from pigs, with different levels of inflammatory response. In contrast, other biomaterials, such as human acellular dermal matrix (HADM) and primate acellular dermal matrix (PADM), also studied by Xu et al. (17), demonstrated more effective tissue integration, accompanied by mild and transient inflammation.

Studies suggested that this polarization can be influenced by any type of implant, including polymeric, ceramic, metallic, and composite biomaterials, depending on their intrinsic characteristics, such as material composition, surface texture, and interactions with the biological environment. For example, Won and colleagues (36) demonstrated that the predominance of M2-type macrophages, associated with a pro-reparative response, contributed to better tissue regeneration in polycaprolactone (PCL) scaffolds. In their study, PCL scaffolds with modified surfaces promoted a higher prevalence of M2 macrophages, along with increased angiogenic factors (such as VEGF), reduced pro-inflammatory chemokines, and decreased fibrous capsule formation.

Similarly, research involving biomaterials based on collagen, chitosan, and other composites, such as the studies by Scaglione et al. (20) and Caires et al. (31), highlighted that the composition of the biomaterial significantly influenced new tissue formation and the associated inflammatory response. Furthermore, Razzi et al. (35), who assessed ceramic materials, and Huang et al. (32), who studied metallic materials, emphasized that physicochemical properties, such as porosity, surface structure, and the controlled release of bioactive factors, play crucial roles in regulating cellular responses and enhancing the regenerative potential of implants.

**Figure 3:**
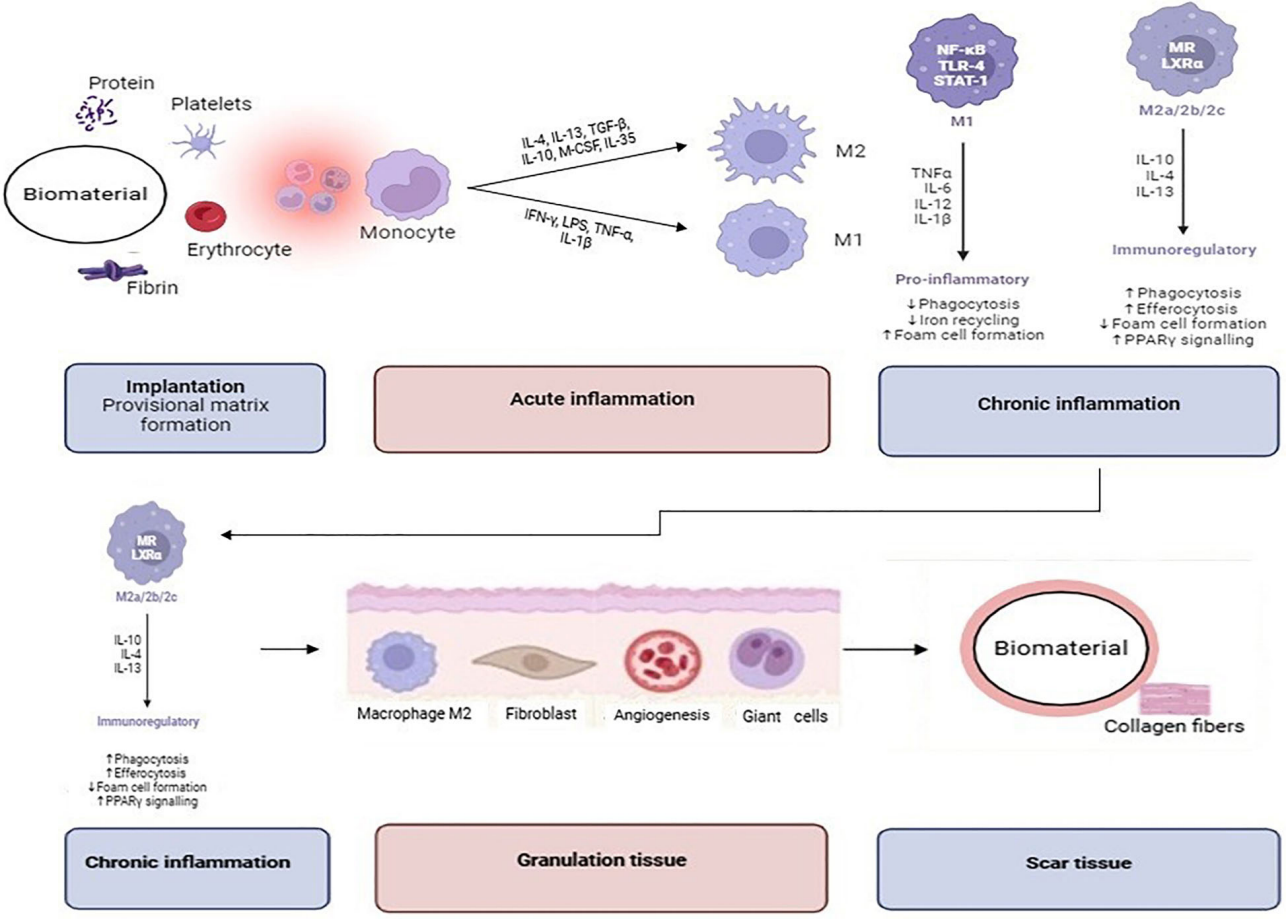
Macrophage polarization and tissue regeneration induced by biomaterials. The figure illustrates the sequence of events following the implantation of a biomaterial, from the formation of a provisional matrix to macrophage polarization into M1 (pro-inflammatory) or M2 (pro-regenerative) phenotypes. M2 macrophages, induced by factors such as IL-10 and TGF-β, promote inflammation resolution, recruitment of mesenchymal stem cells (MSCs), and tissue regeneration. These processes lead to the transition from granulation tissue to effective scar formation. The role of biomaterials in modulating the immune response to support tissue repair is highlighted.

Macrophage polarization stands out as a key factor in modulating the immune response and the healing process (Figure 3). Understanding how the immune system interacts with biomaterials is essential in determining the success or failure of an implant. Interactions between the immune system and the biomaterial trigger a cascade of biological events that can promote efficient implant integration or result in adverse reactions, such as chronic inflammation and material rejection. During the acute phase, M1 macrophages predominate, playing a crucial role in defending against pathogens, clearing cellular debris, and initiating the healing process. As the response progresses, a transition occurs to the M2 profile, which promotes inflammation resolution, tissue repair, and angiogenesis (36).

However, in the chronic phase, a coexistence of M1 and M2 macrophages is observed. M1 macrophages in the chronic phase remain associated with the production of pro-inflammatory cytokines, which can contribute to adverse reactions such as fibrosis or persistent inflammation. On the other hand, M2 macrophages during this phase are essential for sustaining tissue repair by promoting extracellular matrix deposition and modulating the inflammatory response in a beneficial way. The balance between these macrophage subpopulations determines whether the process will result in efficient implant integration or complications related to chronic inflammation. The interaction of host tissue cells with the biomaterial also promotes the migration and proliferation of progenitor cells, such as mesenchymal stem cells, which are essential for tissue regeneration. This regenerative process involves the formation of new tissues, restoration of tissue function, and the effective integration of the biomaterial into the host (30,31).

The biological response to an implanted biomaterial is directly related to its intrinsic properties. Active biomaterials can interact with the surrounding tissue, promoting processes such as angiogenesis, tissue formation, and cellular integration, which often result in a thin or even absent fibrous capsule. However, some biomaterials may trigger a more prolonged inflammatory response, leading to the formation of a thick fibrous capsule that isolates the implant from the tissue. Thus, the formation and thickness of this capsule are determined by the characteristics of the biomaterial used, highlighting the importance of selecting materials appropriate to the specific application needs (32,35).

### Immune system and biomaterial integration

The immune system is crucial in responding to implanted biomaterials, divided into innate and adaptive. The innate system acts as the first line of defense, responding quickly and non-specifically to foreign materials, through physical barriers, macrophages, and complementary proteins.

The adaptive system provides a specific and memory-based response, being activated, when necessary, and involves T and B lymphocytes that recognize specific antigens. This system can play a relevant role in tissue regeneration, where regulatory T cells (Tregs), for example, are associated with promoting tissue regeneration by modulating inflammation and stimulating cell proliferation, and the antibodies produced by B lymphocytes may contribute to the recognition of biomaterial components and influence the tissue response. In the context of biomaterials, this interaction can determine the success or failure of the implant, with a controlled inflammatory response being essential for the adequate integration of the biomaterial (41,42).

Macrophages, dominant effector cells during inflammation, are recruited and activated, and can adopt a pro-inflammatory (M1) or anti-inflammatory and tissue repair (M2) phenotype. M1 activation is triggered by factors such as lipopolysaccharide (LPS) and interferon gamma (IFN-γ), while M2 activation is induced by interleukin 4 (IL-4), interleukin 13 (IL-13), and interleukin 10 (IL-10) (23,33,38,41). M1 macrophages are essential in the initial phase for debridement and disinfection of the injury site, while M2 are crucial for the repair phase (22).

During the remodeling phase, the macrophage population changes from predominantly pro-inflammatory (M1) to predominantly anti-inflammatory (M2), helping to promote angiogenesis and modulate the immune response (20,21). Although the formation of functional blood vessels (angiogenesis) is not directly related to the immune system, it facilitates the mobilization of immune cells to the site of injury, contributing to the immune response and the repair process (25).

The complexity and importance of cellular interactions in the initial phases of the inflammatory response and subsequent tissue regeneration are significant. Phenotypic reprogramming of macrophages, mediated by functional biomaterials, can modulate the inflammatory response, favoring an environment that is more conducive to tissue regeneration (36).

Biomaterial is any substance, of synthetic or natural origin, used temporarily as part or as a whole of a biological system, playing a crucial role in tissue engineering and regenerative medicine. The interaction between biomaterials and the host's immune system is a determining factor for the success of the implant. Inflammatory reactions triggered by biomaterials can be seen as harmful, which directs efforts to minimize them. However, these immune responses are not necessarily harmful (35).

The biological response to biomaterials is mediated by the interaction of the implanted material with the surrounding environment. In the initial phases of implantation, adhesion proteins such as fibronectin and vitronectin adsorb on the surface of the biomaterial, creating a provisional matrix. This is followed by the accumulation of immunocompetent cells that recognize these proteins, which is crucial for the subsequent biological response to implantation. Monocytes and macrophages are recruited to the wound site, driven by chemoattractants such as interleukin 1 beta (IL-1β), interleukin 4 (IL-4), tumor necrosis factor alpha (TNF-α) and the chemokine CCL2, among others, which facilitate cell proliferation and extravasation (43).

The immediate adsorption of proteins onto the surface of the biomaterial triggers a series of events, including the activation of immune cells and the formation of a provisional fibrin matrix. The topography and composition of the biomaterial also influence this response, affecting macrophage polarization and the production of pro-inflammatory and pro-angiogenic cytokines (20,21).

After implantation of biomaterials, the initial inflammatory response is triggered, with neutrophils being the first to migrate to the injury site in the first 24 h. They secrete growth factors, such as vascular endothelial growth factor (VEGF), to promote angiogenesis and stimulate cell proliferation (44). Furthermore, neutrophils can form extracellular traps (NETs), essential for the activation of new immunological and inflammatory responses (36).

Macrophages, myeloid cells derived from monocytes, play a critical role in the inflammatory response. They secrete signaling molecules that regulate cell migration and differentiation, tissue remodeling, and the formation of new blood vessels. Polarization towards an M2 phenotype is crucial to promote an anti-inflammatory action favorable to the integration of the biomaterial (21,37).

During the remodeling phase, macrophages continue to play essential roles. They regulate the inflammatory response and biomaterial integration, influenced by characteristics such as surface chemistry, topography, and hydrophilicity of the biomaterials. This balance is crucial for the acceptance of the biomaterial by the host (35).

The properties of biomaterials, such as roughness, chemical composition, porosity, and hydrophobicity, significantly influence the biological responses during tissue interaction. For example, roughness favors cell adhesion, while chemical composition can affect the polarization of immune cells, modulating inflammatory responses from M1 to M2. Porosity facilitates cell infiltration, and hydrophobicity regulates initial protein adsorption, key aspects for biocompatibility. Table 1 summarizes these characteristics, showing how these properties influence the initial immune response, which involves the activation of the complement system and the recruitment of neutrophils and macrophages. The expected outcomes include improved biocompatibility, cell adhesion, vascularization, and reduced fibrous capsule formation, elements that contribute to the clinical efficiency and tissue integration of biomaterials.

**Table 1:** Characteristics of biomaterials and their biological responses. The table summarizes the key properties of biomaterials, and their respective influences on the initial immune response and expected outcomes.

Characteristics	Description
Properties of biomaterials	
Roughness	Rough surfaces increase cell adhesion.
Chemical composition	Influence on M1/M2 polarization.
Porosity	Favors cell infiltration.
Hydrophobicity	Regulates initial protein adsorption.
Initial immune response	
Activation of the complement system	Activation of the immune defense system.
Recruitment of neutrophils and macrophages	Increase in immune cells at the implantation site.
Expected outcomes	
Biocompatibility	Reduced inflammation.
Tissue integration	Cell adhesion and vascularization.
Reduced fibrous capsule formation	Increased clinical efficiency.

### Specific case studies

In this review, different studies explored the interactions between biomaterials and the biological system in specific contexts, including increased cell infiltration, macrophage polarization from M1 to M2, reduction of pro-inflammatory cytokines, stimulation of angiogenic factors, promotion of osteogenic differentiation, and reduction of fibrotic capsule formation, contributing to a better understanding of immune response modulation, while promoting tissue healing and regeneration. For example, Abebayehu and colleagues (29), demonstrated that with the incorporation of galectin-1 in electrospun polydioxanone (PDO) scaffolds, there was a polarization of macrophages towards the M2 phenotype, favoring tissue regeneration. This demonstrates the ability to manipulate the macrophage immune response to optimize healing.

Corradetti and colleagues (30) investigated the functionalization of a collagen scaffold with chondroitin sulfate in a subcutaneous implant model. Functionalization refers to the process of modifying or enhancing a biomaterial by adding bioactive molecules with the aim of imparting specific properties to the material that improve its interaction with the body, promoting a more favorable biological response. The authors demonstrated that the material was effective in inducing a pro-regenerative environment, characterized by early recruitment and sustained retention of anti-inflammatory macrophages. This immune response modulation reduced the pro-inflammatory environment and promoted efficient healing, preventing chronic inflammation. Scaffold functionalization facilitated remodeling of the extracellular matrix, reorganization of collagen fibrils, formation of blood vessels, and proper integration of the implant with the surrounding tissue, highlighting the potential of functionalized collagen scaffolds for tissue regeneration. Corradetti et al. (30) and Spiller et al. (23) concluded that strategies such as the functionalization of biomaterials with bioactive molecules can help modulate the immune response and promote tissue regeneration.

The choice of specific biomaterials significantly impacts the biological response due to their composition, structural properties, and interaction with the host's immune system. Studies conducted by Sandor et al. (16) and Xu et al. (17) evaluated the use of porcine-derived biological meshes for abdominal wall resection repair in primates, observing distinct mechanisms of rejection or integration. For example, the small intestinal submucosa triggered a significant inflammatory response and scar tissue formation due to the presence of residual antigenic components that activated the host's immune system. Porcine dermal grafts showed minimal integration, accompanied by a pronounced cellular immune response, likely attributed to incomplete decellularization, which left immunogenic elements that impaired proper tissue remodeling. On the other hand, the acellular dermal matrix (HADM) demonstrated a more favorable biological response, attributed to its complete decellularization process, which removed immunogenic components while preserving the extracellular matrix structure. This facilitated early cell repopulation, the formation of functional blood vessels, and better integration with surrounding tissues, highlighting its potential for clinical applications in tissue repair and regeneration.

Vasconcelos and colleagues (27) investigated the use of fibrinogen scaffolds (Fg-3D) to repair bone defects in rats, highlighting their ability to promote a pro-regenerative response. The biomaterial facilitated defect closure by providing a 3D matrix that favored cell adhesion and the migration of mesenchymal stem cells, stimulating bone regeneration. Additionally, the presence of the scaffold influenced the inflammatory response, increasing the population of immune cells at the defect site. This indicated favorable immune modulation, promoting a transition from an initial inflammatory response to an anti-inflammatory and pro-regenerative profile. This balance was crucial to prevent chronic inflammation and ensure proper biomaterial integration, suggesting the potential of fibrinogen scaffolds in bone regeneration and immune response modulation.

The studies by Mahmoudzadeh et al. (26) and López-Dolado et al. (25) investigated the importance of the topography and three-dimensional structure of biomaterials in modulating cellular responses, especially interactions with macrophages. Mahmoudzadeh et al. (26) observed that 3D collagen and chitosan scaffolds provided a more favorable environment for macrophage function and activation, with increased phagocytic activity and production of pro-inflammatory cytokines (TNF and IL-1). Additionally, the uptake of chitosan nanoparticles by macrophages was more efficient in the 3D scaffolds, suggesting that this structure facilitates cellular interaction with the biomaterial. López-Dolado et al. (25), in an *in vivo* model with Wistar rats and graphene oxide scaffolds, observed a favorable immune response, characterized by the presence of cells expressing activation markers (GFAP) and increased cell density and collagen fiber formation. This study also showed a decrease in inflammatory cells, indicating that the biomaterial helped reduce chronic inflammation and favored tissue regeneration. Both studies highlighted the importance of 3D biomaterial architecture, as it not only facilitates cellular activation and immune response but also promotes tissue regeneration, encouraging the formation of blood vessels and tissue integration with the biomaterial, which is essential for successful tissue repair and regeneration.

Yang and colleagues (38) investigated the impact of the micro/nanotextured topography of biomaterials, such as the Ti-6Al-4V alloy, on the cellular response. The study showed that this topography promoted the polarization of macrophages towards the M2 phenotype, resulting in an anti-inflammatory effect, which is essential for tissue regeneration. Additionally, it facilitated the migration, proliferation, and mineralization of osteoblasts, key cells in bone regeneration. The increase in proteins such as BMP2 and VEGF was observed, highlighting the induction of bone formation and angiogenesis. These results suggested that the biomaterial's topography regulated the cellular response and effectively promoted bone regeneration, favoring implant integration with tissue.

Biomaterials with channelized microstructures induced biological responses that were fundamental for tissue regeneration, including the modulation of immunological and inflammatory reactions, promotion of angiogenesis and stimulation of stem cell recruitment. These favorable interactions highlighted the potential of microchannels to accelerate tissue regeneration, including the repair of defective bones, paving the way for significant advances in tissue engineering and regenerative medicine, as studied by Won et al. (36).

Another key aspect is the interaction of biomaterial surfaces and osteoimmunomodulation. Modifying biomaterial surfaces, such as applying plasma electrolytic oxidation (PEO) to titanium implants, can promote osteoimmunomodulation and antibacterial functionalities, improving implant acceptance and preventing infections. Additionally, the cellular response to biomaterials may vary depending on the surface conditions of the implants. Human primary macrophages react differently to titanium implants depending on the surface conditions of the materials. This occurs because macrophages, as key cells in the immune response and healing process, interact directly with the biomaterial surface, detecting characteristics such as roughness, chemical composition, and surface energy. These interactions determine whether the macrophages adopt a pro-inflammatory (M1) profile, which can lead to implant rejection, or an anti-inflammatory and pro-regenerative (M2) profile, which favors tissue integration and regeneration. As observed by Razzi et al. (35), surface treatments, such as plasma electrolytic oxidation (PEO), can modulate this cellular response by promoting greater biocompatibility and specific functionalities, such as antibacterial properties.

Bone regeneration and vascularization are complex processes that involve the dynamic interaction between biomaterials and the biological environment. In the context of bone repair, the initial inflammatory process plays a critical role in promoting revascularization and recruitment of osteoprogenitor cells to the fracture site. The formation of new blood vessels and early cell repopulation are observed, with fibroblasts infiltrating the edge of the material and the formation of functional vessels lined by endothelial cells at the end of the first month, suggesting effective integration and a favorable cellular response (24).

Finally, it is particularly interesting to note that in this review, studies investigated different strategies to modulate the immune response in biomaterials. Vigneswaran et al. (28) reported that peptide biomaterials, despite being highly immunogenic, did not impair normal wound healing, being promising for the development of biomaterials in tissue engineering and wound repair. Studies by Keane et al. (21) and Scaglione et al. (20), highlighted that the functionalization of biomaterials promoted the polarization of macrophages towards the M2 phenotype, favoring healing and tissue regeneration. This results in efficient remodeling of the extracellular matrix, reorganization of collagen fibrils, and formation of new blood vessels, which are essential for the structural integrity of the regenerated tissue.

Caires et al. (31) investigated the effect of polylactic acid (PLA) and chitosan biomaterials on cytokine production and cell recruitment, particularly of dermal fibroblasts (HDF) and mesenchymal stem cells (MSCs). They observed that chitosan, when used in scaffolds, increased fibroblast recruitment, suggesting a potential to induce a more pronounced fibrotic response, beneficial for connective tissue regeneration. However, in the presence of MSCs, fibroblast recruitment was reduced, indicating that MSCs may modulate the fibrogenic response and promote a more balanced tissue regeneration.

Microribbon (μRB) scaffolds coated with mesenchymal stem cell (MSCM) membranes studied by Fu et al. (39) were shown to induce both innate and adaptive regenerative immune responses in a critical-sized cranial defect model. The presence of the MSC membrane was shown to promote macrophage polarization toward a regenerative phenotype and control CD8+ T cell apoptosis, while encouraging regulatory T cell (Treg) differentiation, culminating in accelerated bone regeneration. Combining the scaffolds with a low dose of BMP-2 not only boosted bone regeneration but also reduced inflammation associated with high-dose BMP-2, representing a promising strategy for tissue bioengineering and healing of critical bone defects.

Based on the studies analyzed, this review demonstrates significant advancements in biomaterials research, particularly in understanding and modulating the immune response to improve tissue regeneration and healing. These efforts aim to achieve optimal interactions between the tissue and the implant, promoting more favorable outcomes in tissue integration. Variations in immune responses were observed based on the physicochemical properties and topographies of biomaterials, reinforcing the importance of material composition and structural features in influencing host interactions.

Bioactive materials demonstrated superior potential to facilitate tissue regeneration, while inert materials provoked moderate inflammatory reactions, with macrophage polarization playing a central role in modulating the immune response. Advances in surface modifications, such as micro/nanotexturing and plasma electrolytic oxidation, have also enhanced the osteoimmunomodulatory and antibacterial properties of implants, contributing to improved clinical outcomes. The strategic manipulation of immune responses through biomaterial design, including macrophage polarization and regulatory T cell differentiation, offers promising pathways to enhance integration and functionality in tissue engineering applications.

These findings not only advance the understanding of interactions between biomaterials and tissues but also provide critical insights to guide future research. Investigating more targeted strategies for immune modulation, such as the precise tuning of material properties to achieve specific macrophage and T cell responses, holds the potential to revolutionize regenerative medicine. Clinically, this knowledge paves the way for developing personalized implants tailored to the specific immunological profiles of patients, reducing complications and increasing therapeutic success across various applications, from orthopedics to soft tissue repair.

The evidence strongly supports the need for personalized approaches in biomaterial selection, focusing on biocompatibility and long-term tissue performance to optimize therapeutic efficacy in clinical settings.

## Supplementary Material

Click to view [pdf].

## References

[1] Naidu NA, Wadher K, Umekar M (2021). An overview on biomaterials: pharmaceutical and biomedical applications. J Drug Deliv Ther.

[2] Trucillo P (2024). Biomaterials for drug delivery and human applications. Materials.

[3] Brokesh AM, Gaharwar AK (2020). Inorganic biomaterials for regenerative medicine. ACS Appl Mater Interfaces.

[4] Abaricia JO, Farzad N, Heath TJ, Simmons J, Morandini L, Olivares-Navarrete R (2021). Control of innate immune response by biomaterial surface topography, energy, and stiffness. Acta Biomater.

[5] Zhang B, Su Y, Zhou J, Zheng Y, Zhu D (2021). Toward a better regeneration through implant-mediated immunomodulation: harnessing the immune responses. Adv Sci (Weinh).

[6] Dee KC, Puleo DA, Bizios R (2002). An introduction to tissue-biomaterial interactions.

[7] Li R, Liu K, Huang X, Li D, Ding J, Liu B (2022). Bioactive materials promote wound healing through modulation of cell behaviors. Adv Sci (Weinh).

[8] Balabiyev A, Podolnikova NP, Kilbourne JA, Baluch DP, Lowry D, Zare A (2021). Fibrin polymer on the surface of biomaterial implants drives the foreign body reaction. Biomaterials.

[9] Hernandez JL, Park J, Yao S, Blakney AK, Nguyen HV, Katz BH (2021). Effect of tissue microenvironment on fibrous capsule formation to biomaterial-coated implants. Biomaterials.

[10] Amin Yavari S, Castenmiller SM, van Strijp JA, Croes M (2020). Combating implant infections: shifting focus from bacteria to host. Adv Mater.

[11] Kämmerling L, Fisher LE, Antmen E, Simsek GM, Rostam HM, Vrana NE (2021). Mitigating the foreign body response through ‘immune-instructive' biomaterials. J Immunol Regener Med.

[12] Babykutty S, Suboj P, Udayan S (2024). Introduction to immune responses toward medical implants.

[13] Page MJ, McKenzie JE, Bossuyt PM, Boutron I, Hoffmann TC, Mulrow CD (2023). The PRISMA 2020 statement: an updated guideline for reporting systematic reviews [in Portuguese]. Rev Panam Salud Publica.

[14] Schardt C, Adams MB, Owens T, Keitz S, Fontelo P (2007). Utilization of the PICO framework to improve searching PubMed for clinical questions. BMC Med Inform Decis Mak.

[15] Moher D, Liberati A, Tetzlaff J, Altman DG, PRISMA Group (2010). Preferred reporting items for systematic reviews and meta-analyses: the PRISMA statement. Int J Surg.

[16] Sandor M, Xu H, Connor J, Lombardi J, Harper JR, Silverman RP (2008). Host response to implanted porcine-derived biologic materials in a primate model of abdominal wall repair. Tissue Eng Part A.

[B17] Xu H, Wan H, Sandor M, Qi S, Ervin F, Harper JR (2008). Host response to human acellular dermal matrix transplantation in a primate model of abdominal wall repair. Tissue Eng Part A.

[B18] Soder BL, Propst JT, Brooks TM, Goodwin RL, Friedman HI, Yost MJ (2009). The connexin43 carboxyl-terminal peptide ACT1 modulates the biological response to silicone implants. Plast Reconstr Surg.

[B19] Joseph J, Mohanty M, Mohanan PV (2010). Role of immune cells and inflammatory cytokines in regulation of fibrosis around silicone expander implants. J Mater Sci Mater Med.

[B20] Scaglione S, Cilli M, Fiorini M, Quarto R, Pennesi G (2011). Differences in chemical composition and internal structure influence systemic host response to implants of biomaterials. Int J Artif Organs.

[B21] Keane TJ, Londono R, Turner NJ, Badylak SF (2012). Consequences of ineffective decellularization of biologic scaffolds on the host response. Biomaterials.

[B22] Bryan N, Ashwin H, Smart N, Weyhe D, Wohlert S, Bayon Y (2015). Systemic inflammatory cytokine analysis to monitor biomaterial augmented tissue healing. Int J Artif Organs.

[B23] Spiller KL, Nassiri S, Witherel CE, Anfang RR, Ng J, Nakazawa KR (2015). Sequential delivery of immunomodulatory cytokines to facilitate the M1-to-M2 transition of macrophages and enhance vascularization of bone scaffolds. Biomaterials.

[B24] Graney PL, Roohani-Esfahani SI, Zreiqat H, Spiller KL (2016). *In vitro* response of macrophages to ceramic scaffolds used for bone regeneration. J R Soc Interface.

[B25] López-Dolado E, González-Mayorga A, Gutiérrez MC, Serrano MC (2016). Immunomodulatory and angiogenic responses induced by graphene oxide scaffolds in chronic spinal hemisected rats. Biomaterials.

[B26] Mahmoudzadeh A, Mohsenifar A, Rahmani-Cherati T (2016). Collagen-chitosan 3-D nano-scaffolds effects on macrophage phagocytosis and pro-inflammatory cytokine release. J Immunotoxicol.

[B27] Vasconcelos DM, Gonçalves RM, Almeida CR, Pereira IO, Oliveira MI, Neves N (2016). Fibrinogen scaffolds with immunomodulatory properties promote *in vivo* bone regeneration. Biomaterials.

[B28] Vigneswaran Y, Han H, De Loera R, Wen Y, Zhang X, Sun T (2016). This paper is the winner of an SFB Award in the Hospital Intern, Residency category: peptide biomaterials raising adaptive immune responses in wound healing contexts. J Biomed Mater Res A.

[B29] Abebayehu D, Spence A, Boyan BD, Schwartz Z, Ryan JJ, McClure MJ (2017). Galectin-1 promotes an M2 macrophage response to polydioxanone scaffolds. J Biomed Mater Res A.

[B30] Corradetti B, Taraballi F, Corbo C, Cabrera F, Pandolfi L, Minardi S (2017). Immune tuning scaffold for the local induction of a pro-regenerative environment. Sci Rep.

[B31] Caires HR, da Silva PB, Barbosa MA, Almeida CR (2018). A co-culture system with three different primary human cell populations reveals that biomaterials and MSC modulate macrophage-driven fibroblast recruitment. J Tissue Eng Regen Med.

[B32] Huang Y, Wu C, Zhang X, Chang J, Dai K (2018). Regulation of immune response by bioactive ions released from silicate bioceramics for bone regeneration. Acta Biomater.

[B33] Li M, Gao L, Chen J, Zhang Y, Wang J, Lu X (2018). Controllable release of interleukin-4 in double-layer sol-gel coatings on TiO(2) nanotubes for modulating macrophage polarization. Biomed Mater.

[B34] Shu Y, Yu Y, Zhang S, Wang J, Xiao Y, Liu C (2018). The immunomodulatory role of sulfated chitosan in BMP-2-mediated bone regeneration. Biomater Sci.

[B35] Razzi F, Fratila-Apachitei L, Fahy N, Bastiaansen-Jenniskens YM, Apachitei I, Farrell E (2020). Immunomodulation of surface biofunctionalized 3D printed porous titanium implants. Biomed Mater.

[B36] Won JE, Lee YS, Park JH, Lee JH, Shin YS, Kim CH (2020). Hierarchical microchanneled scaffolds modulate multiple tissue-regenerative processes of immune-responses, angiogenesis, and stem cell homing. Biomaterials.

[B37] Kazimierczak P, Koziol M, Przekora A (2021). The Chitosan/Agarose/NanoHA bone scaffold-induced M2 macrophage polarization and its effect on osteogenic differentiation *in vitro*. Int J Mol Sci.

[B38] Yang Y, Zhang T, Jiang M, Yin X, Luo X, Sun H (2021). Effect of the immune responses induced by implants in a integrated three-dimensional micro-nano topography on osseointegration. J Biomed Mater Res A.

[B39] Fu M, Li J, Liu M, Yang C, Wang Q, Wang H (2023). Sericin/Nano-hydroxyapatite hydrogels based on graphene oxide for effective bone regeneration via immunomodulation and osteoinduction. Int J Nanomedicine.

[40] Su N, Villicana C, Barati D, Freeman P, Luo Y, Yang F (2023). Stem cell membrane-coated microribbon scaffolds induce regenerative innate and adaptive immune responses in a critical-size cranial bone defect model. Adv Mater.

[41] Leifer CA (2017). Dendritic cells in host response to biologic scaffolds. Semin Immunol.

[42] Mariani E, Lisignoli G, Borzì RM, Pulsatelli L (2019). Biomaterials: foreign bodies or tuners for the immune response?. Int J Mol Sci.

[43] Geelhoed WJ, Moroni L, Rotmans JI (2017). Utilizing the foreign body response to grow tissue engineered blood vessels *in vivo*. J Cardiovasc Transl Res.

[44] Yang D, Xiao J, Wang B, Li L, Kong X, Liao J (2019). The immune reaction and degradation fate of scaffold in cartilage/bone tissue engineering. Mater Sci Eng C Mater Biol Appl.

