# Supplementary material for: Biological responses to biomaterials: a review
Source: Braz J Med Biol Res. 2025 May 9;58:e14599. doi: 10.1590/1414-431X2025e14599 (PMC12068782; doi:10.1590/1414-431X2025e14599)
Supplement: Supplementary file 1 [file 1414-431X-bjmbr-58-e14599-suppl.pdf]

Table S1. Search strategies used for each database.

Database	String	Results	Search date
BVS	(mh:"Próteses e Implantes" OR (Próteses e Implantes) OR (Prostheses and Implants) OR (Prótesis e Implantes) OR (Prothèses et implants) OR (Endoprótese) OR (Endopróteses) OR (Implantação de Próteses) OR (Implante Protético) OR (Implante de Prótese) OR (Implantes Artificiais) OR (Implantes de Próteses) OR (Prótese) OR (Próteses) OR (Próteses e Implante) OR mh:E07.695$ OR mh:VS2.006.002.010$) AND (mh:"Regeneração" OR (Regeneração) OR (Regeneration) OR (Regeneración) OR (Régénération) OR (Regeneração Endógena) OR mh:G16.762$) AND (mh:"Fatores Imunológicos" OR (Fatores Imunológicos) OR (Immunologic Factors) OR (Factores Inmunológicos) OR (Facteurs immunologiques) OR (Biomodulador) OR (Fator Imune) OR (Fator Imunológico) OR (Imunomodulador) OR (Imunomoduladores) OR (Modificador da Resposta Biológica) OR (Modificador de Resposta Biológica) OR (Modificadores da Resposta Biolológica) OR (Modificadores de Resposta Biolológica) OR mh:D27.505.696.477$) AND (mh:"Materiais Biocompatíveis" OR (Materiais Biocompatíveis) OR (Biocompatible Materials) OR (Materiales Biocompatibles) OR (Matériaux biocompatibles) OR (Biomateriais) OR (Biomaterial) OR (Materiais Bioartificiais) OR (Material Bioartificial) OR (Material Biocompatível) OR (Material Biológico) OR (Material Hemocompatível) OR mh:D25.130$ OR mh:D27.720.102.130$ OR mh:J01.637.051.130$)	76	29.08.2024
PubMed	((("Prostheses and Implants"[Mesh] OR (Implants and Prostheses) OR (Prosthetic Implants) OR (Prostheses and Implant) OR (Implant and Prostheses) OR (Prosthetic Implant) OR (Implant, Prosthetic) OR (Implants, Prosthetic) OR (Endoprosthesis) OR (Endoprostheses) OR (Prostheses) OR (Prosthesis) OR (Implants, Artificial) OR (Artificial Implant) OR (Artificial Implants) OR (Implant, Artificial)) AND ("Regeneration"[Mesh] OR (Regenerations) OR (Endogenous Regeneration) OR (Regeneration, Endogenous))) AND ("Immunologic Factors"[Mesh] OR (Immunological Factors) OR (Factors, Immunological) OR (Factors, Immunologic) OR (Immune Factors) OR (Factors, Immune) OR (Immune Factor) OR (Factor, Immune) OR (Immunological Factor) OR (Factor, Immunological) OR (Immunomodulators) OR (Immunomodulator) OR (Biological Response Modifiers) OR (Biological Response Modifier) OR (Modifier, Biological Response) OR (Response Modifier, Biological) OR (Biomodulators) OR (Response Modifiers, Biological) OR (Biomodulator) OR (Modifiers, Biological Response))) AND ("Biocompatible Materials"[Mesh] OR (Biocompatible Material) OR (Material, Biocompatible) OR (Biomaterials) OR (Biomaterial) OR (Bioartificial Materials) OR (Bioartificial Material) OR (Material, Bioartificial) OR (Hemocompatible Materials) OR (Hemocompatible Material) OR (Material, Hemocompatible))	584	29.08.2024
Embase	('prostheses and orthoses'/exp OR 'prostheses and implants' OR 'prostheses and orthoses') AND ('regeneration'/exp OR 'regeneration process' OR 'regenerative activity' OR 'regeneration') AND ('immunologic factor'/exp OR 'immunologic factors' OR 'immunologic factor') AND ('biomaterial'/exp OR 'biocompatible materials' OR 'biologic material' OR 'biological material' OR 'biomaterial')	1	29.08.2024
Scopus	(ALL (("Prostheses and Implants" OR "Implants and Prostheses" OR "Prosthetic Implants" OR "Prostheses and Implant" OR "Implant and Prostheses" OR "Prosthetic Implant" OR "Implant, Prosthetic" OR "Implants, Prosthetic" OR "Endoprosthesis" OR "Endoprostheses" OR "Prostheses" OR "Prosthesis" OR "Implants, Artificial" OR "Artificial Implant" OR "Artificial Implants" OR "Implant, Artificial")) AND ALL (("Regeneration" OR "Regenerations" OR "Endogenous Regeneration" OR "Regeneration, Endogenous")) AND ALL (("Immunologic Factors" OR "Immunological Factors" OR "Factors, Immunological" OR "Factors, Immunologic" OR "Immune Factors" OR "Factors, Immune" OR "Immune Factor" OR "Factor, Immune" OR "Immunological Factor" OR "Factor, Immunological" OR "Immunomodulators" OR "Immunomodulator" OR "Biological Response Modifiers" OR "Biological Response Modifier" OR "Modifier, Biological Response" OR "Response Modifier, Biological" OR "Biomodulators" OR "Response Modifiers, Biological" OR "Biomodulator" OR "Modifiers, Biological Response")) AND ALL (("Biocompatible Materials" OR "Biocompatible Material" OR "Material, Biocompatible" OR "Biomaterials" OR "Biomaterial" OR "Bioartificial Materials" OR "Bioartificial Material" OR "Material, Bioartificial" OR "Hemocompatible Materials" OR "Hemocompatible Material" OR "Material, Hemocompatible")))	111	29.08.2024
Web of science	(Prostheses and Implants* OR Implants and Prostheses* OR Prosthetic Implants* OR Prostheses and Implant* OR Implant and Prostheses* OR Prosthetic Implant* OR Implant, Prosthetic* OR Implants, Prosthetic* OR Endoprosthesis* OR Endoprostheses* OR Prostheses* OR Prosthesis* OR Implants, Artificial* OR Artificial Implant* OR Artificial Implants* OR Implant, Artificial*) AND (Regeneration* OR Regenerations* OR Endogenous Regeneration* OR Regeneration, Endogenous*) AND (Immunologic Factors* OR Immunological Factors* OR Factors, Immunological* OR Factors, Immunologic* OR Immune Factors* OR Factors, Immune* OR Immune Factor* OR Factor, Immune* OR Immunological Factor* OR Factor, Immunological* OR Immunomodulators* OR Immunomodulator* OR Biological Response Modifiers* OR Biological Response Modifier* OR Modifier, Biological Response* OR Response Modifier, Biological* OR Biomodulators* OR Response Modifiers, Biological* OR Biomodulator* OR Modifiers, Biological Response*) AND (Biocompatible Materials* OR Biocompatible Material* OR Material, Biocompatible OR Biomaterials* OR Biomaterial* OR Bioartificial Materials* OR Bioartificial Material* OR Material, Bioartificial* OR Hemocompatible Materials* OR Hemocompatible Material* OR Material, Hemocompatible*)	19	29.08.2024

Table S2. Criteria of exclusion of studies.

Author/year	Criteria of exclusion
DeLustro et al. 1990	3
Babensee et al. 1998	3
Endres et al. 2005	2
Kyung et al. 2007	1
Boehler et al. 2011	3
Franz et al. 2011	3
Lieder et al. 2013	3
Santos et al. 2013	1
Tan et al. 2013	1
Ballotta et al. 2014	2
Seebach et al. 2014	2
Bassi et al. 2015	2
Choe et al. 2015	1
Sheikh et al. 2015	3
Albrektsson et al. 2016	2
Hortensius et al. 2016	2
Liu et al. 2016	2
Dziki et al. 2017	3
Geelhoed et al. 2017	3
Leifer, 2017	3
Olingy et al. 2017	3
Shi et al. 2017	2
Yang et al. 2017	2
Biguetti et al. 2018	2
Zheng et al. 2018	2
Chen et al. 2019	3
Kanjevac et al. 2019	3
Mariani et al. 2019	2
Yang et al. 2019	2
Yang et al. 2019	3
Wang et al. 2020	2
Yu et al. 2020	2
Choi et al. 2021	2
Ding et al. 2021	2
El Waly et al. 2021	2
Kiran et al. 2021	3
Li et al. 2021	2
Soni e Rodell, 2021	3
Stojanović et al. 2021	2
Tan et al. 2021	3
Tian et al. 2021	2
Whitaker et al. 2021	3
Zhang et al. 2021	3
Shang et al. 2022	3
Wang et al. 2022	2
Wu et al. 2022	2
Halperin-Sternfeld et al. 2023	2
Ibrahim et al. 2023	3
Ma et al. 2024	2

1: Different intervention: studies that did not involve the use of biomaterials; 2: Different outcome: studies that did not address immune responses (innate or adaptive); 3: Different type of publication: review articles, book chapters, theses, letters, personal opinions, conference abstracts, and patents.

Table S3. Characteristics of the studies included in the review.

Study	Population	Intervention	Outcome
Author, Year/Country, Reference	Model	Biomaterial	Results
Sandor et al. 2008/USA (16)	*In vivo*: vervets caribenhos adultos (3–6 kg) (*C. aetiopes*) (n=33).	Three commercially available swine-derived biological meshes.	↑ Inflammatory responses; ↑ IgG and anti-a-gal antibodies; SIS allowed cellular infiltration, while G-PDM remained acellular.
Xu et al. 2008/USA (17)	*In vivo*: adult male vervet monkeys, weighing 3 to 6 kg (n=73).	Human acellular dermal matrix (HADM), primate acellular dermal matrix (PADM), and a human cellular dermal matrix (HCDM).	HADM did not cause significant chronic inflammatory response; Both HADM and PADM integrated well into the surrounding tissue, with a healthy appearance and no signs of laxity or herniation; ↑ Healing strength between the graft and host tissue; HADM and PADM elicited a mild and transient inflammatory response, whereas HCDM generated a significant inflammatory response.
Soder et al. 2009/USA (18)	*In vivo*: male Harlan Sprague-Dawley rats (200 to 300 g) (n=30).	Silicone discs.	↓ Neutrophil infiltration at 24 and 48 h, and after 2 weeks, ↑ vascularization and ↓ contractile fibroblasts in the capsular tissue. After 4 weeks, ↓ amount of type I collagen and ↓ presence of myofibroblasts.
Joseph et al. 2010/India (19)	*In vivo*: Wistar rats (n=48).	Silicone expander.	After 180 days, a thick fibrous capsule formed around the silicone implant; macrophages and lymphocytes were intensely infiltrated within the first 30 days; ↓ fibroblasts over time, ↑ myofibroblasts around the silicone; ↑ pro-fibrotic cytokines (TGFβ), ↓ anti-fibrotic cytokines (TNFα).
Scaglione et al. 2011/Italy (20)	*In vivo*: Balb/c mice between five and eight weeks of age (n=20).	Hydroxyapatite (HA); Poly-e-Caprolactone (PCL); Type I Collagen (Col).	↑ Circulating blood cells; ↑ MCP-1, IL-5, and FGF-2; Host response varied depending on the biomaterial used, with ↓ FGF-2.
Keane et al. 2012/USA (21)	*In vivo*: Adult female Sprague-Dawley rats (300 g) (n=12).	Extracellular Matrix (ECM) derived from porcine intestinal submucosa (SIS-ECM).	↑ Macrophages, the biomaterial treated with PBS showed ↑ cellular infiltration and ↑ M1 macrophages; after 28 days, ↓ infiltrating cells.
Bryan et al. 2015/UK (22)	*In vivo*: male Wistar rats, 6 weeks old (250 to 270 grams) (n=not specified).	Polyester mesh; Polypropylene mesh with oligocaprone film and polydioxanone glue layer; Polypropylene mesh with sodium hyaluronate/carboxymethyl cellulose film.	↑ MCP-1 and IL-18 in Polyester Mesh.
Spiller et al. 2015/USA (23)	*In vivo*: C57BL/6 mice (n=not specified).	Decellularized bone scaffold prepared from trabecular bone of young cattle.	The sequential release of immunomodulatory cytokines can influence macrophage polarization and, consequently, the vascularization of bone scaffolds.
Graney et al. 2016/USA and Australia (24)	*In vitro*: non-activated macrophages (M0).	Ceramic scaffolds of baghdadite ($Ca_3ZrSi_2O_9$) and strontium-hardystonite-gahnite (Sr-$Ca_2ZnSi_2O_7$-$ZnAl_2O_4$) and tricalcium phosphate-hydroxyapatite (TCP-HA).	Differentiation of macrophage phenotype, ↑ M1 and ↓ M2a and M2c.
López-Dolado et al. 2016/Spain (25)	*In vivo:* Adult male Wistar rats (400 g) (n=14).	Graphene oxide scaffold.	The scaffolds induced a favorable immune response, with the presence of cells expressing activation markers, such as glial fibrillary acidic protein (GFAP); ↑ cell density and collagen fiber formation; ↓ presence of inflammatory cells.
Mahmoudzadeh et al. 2016/Iran (26)	*In vitro*: mouse peritoneal macrophages.	3-D collagen and chitosan scaffold.	↑ Phagocytic activity of macrophages and TNF and IL-1 in 3-D scaffolds; ↑ uptake of chitosan nanoparticles (NP) by macrophages cultured in 3-D scaffolds.
Vasconcelos et al. 2016/Portugal (27)	*In vivo:* male rats of the species *Rattus norvegicus* (Wistar rats) (n=17).	Fibrinogen scaffold.	↑ Immune cell population; the presence of the biomaterial may influence the inflammatory response.
Vigneswaran et al. 2016/USA (28)	*In vivo*: C57BL/6 mice (n=18).	Peptide nanofibers.	↑ Immune response and healing; ↑ CD4 in wound beds; the effect of inflammatory adjuvants, such as CFA and IFA, resulted in an immune response that delayed wound healing.
Abebayehu et al. 2017/USA (29)	*In vitro:* bone marrow-derived macrophages from C57BL/6 mice.	Polydioxanone (PDO) scaffolds.	↑ M2-type macrophage response; ↑ arginase-1.

Corradetti et al. 2017/USA, Italy and UK (30)	*In vivo:* adult Lewis rats (n=not specified).	Chondroitin sulfate functionalized collagen scaffold (CSCL).	↑ IL-10, CD206, iNOS, CD31; ↑ tissue healing, ↓ inflammation and promoting macrophage recruitment.
Caires et al. 2018/ Portugal (31)	*In vitro:* mesenchymal stem cells (MSC); dermal fibroblasts (HDF) and immune cells.	Polylactic acid (PLA) and chitosan.	↑ fibroblasts on chitosan scaffolds; in the presence of MSC ↓ fibroblast recruitment.
Huang et al. 2018/China (32)	*In vitro:* RAW264.7 macrophages. *In vivo*: C57BL/6J mice (n=30).	Akermanitis (AKT); Nagelschmidtite (NAGEL) and -Tricalcium Phosphate (-TCP).	↑ Inflammatory responses; ↓ viability, proliferation and inflammatory cytokines in AKT and NAGEL; ↓ in foreign body giant cell formation *in vivo* on AKT and NAGEL bioceramics compared to -TCP.
Li et al. 2018/China (33)	*In vitro:* murine RAW 264.7 macrophages and Sprague-Dawley rat osteoblasts.	TiO2 nanotubes (TNTs) with surface treatment with carboxymethylated chitosan hydrogel (CMCS).	↓ IL-4 release in GP/IL 4/TNT compared with IL4/TNTs; macrophages switched from M1 to M2 over time; ↑ expression of M2 markers in GP/IL4/TNTs.
Shu et al. 2018/China and Australia (34)	*In vitro:* murine peritoneal macrophages (PMs) and rat bone marrow stromal cells (BMSCs). *In vivo:* female C57/BL mice (n=14).	Chitosan sulfate 2-N,6-O (26SCS).	↑ Inflammatory response; ↓ TNF-α and IL-1β; ↑ osteogenic differentiation of bone marrow stromal cells (BMSCs); ↑ expression of BMP receptors (BMPR-IA, BMPR-IB and BMPR-II).
Razzi et al. 2020/Netherlands (35)	*In vitro:* human monocyte-derived macrophages and human mesenchymal stem cells (hMSCs).	Ti-6Al-4V alloy.	↑ Pro-inflammatory cytokines (IL-6, TNF-α, IL-1β) in untreated implants (SLM NT) and ↓ in treated implants; ↑ polarization towards a pro-repair macrophage phenotype (M2) in treated implants.
Won et al. 2020/South Korea (36)	*In vivo:* C57BL/6 mice and Sprague-Dawley rats (n=not specified).	Poly(caprolactone) (PCL) scaffolds.	Predominance of M2 macrophages in the µCh group, while control scaffolds showed dominant M1 macrophages; ↑ angiogenic factors (VEGF); ↓ pro-inflammatory chemokines; ↓ fibrous capsule formation.
Kazimierczak et al. 2021/Poland (37)	*In vitro*: macrophages, bone marrow-derived mesenchymal stem cells (BMDs) and human osteoblasts (hFOB 1.19).	Chitosan/agarose/nanohydroxyapatite (chit/aga/HA) scaffold.	↑ IL-4, IL-10, and TGF-β; ↑ Bone alkaline phosphatase (bALP) and type I collagen (Col I).
Yang et al. 2021/China (38)	*In vitro*: MC3T3-E1 and RAW264.7 cells.	Ti-6Al-4V alloy.	↑ Cell adhesion and osteoblast proliferation; ↑ BMP2 and VEGF.
Fu et al. 2023/China (39)	*In vivo*: Wistar rats (n=not specified).	Nanocomposite hydrogel composed of alginate (Alg), graphene oxide (GO), sericin (Ser), and nano-hydroxyapatite (nHAP).	↑ Macrophages to the M2 type; ↑ osseointegration at the bone-implant interface; ↑ osteogenic differentiation of bone marrow stem cells (BMSCs); ↓ local inflammation.
Su et al. 2023/USA (40)	*In vitro*: mesenchymal stem cells (MSCs)	Microribbon scaffold (µRB)	↓ Bone formation and mineralization of mesenchymal stem cells (MSCs); ↑ amount of bone formation.
